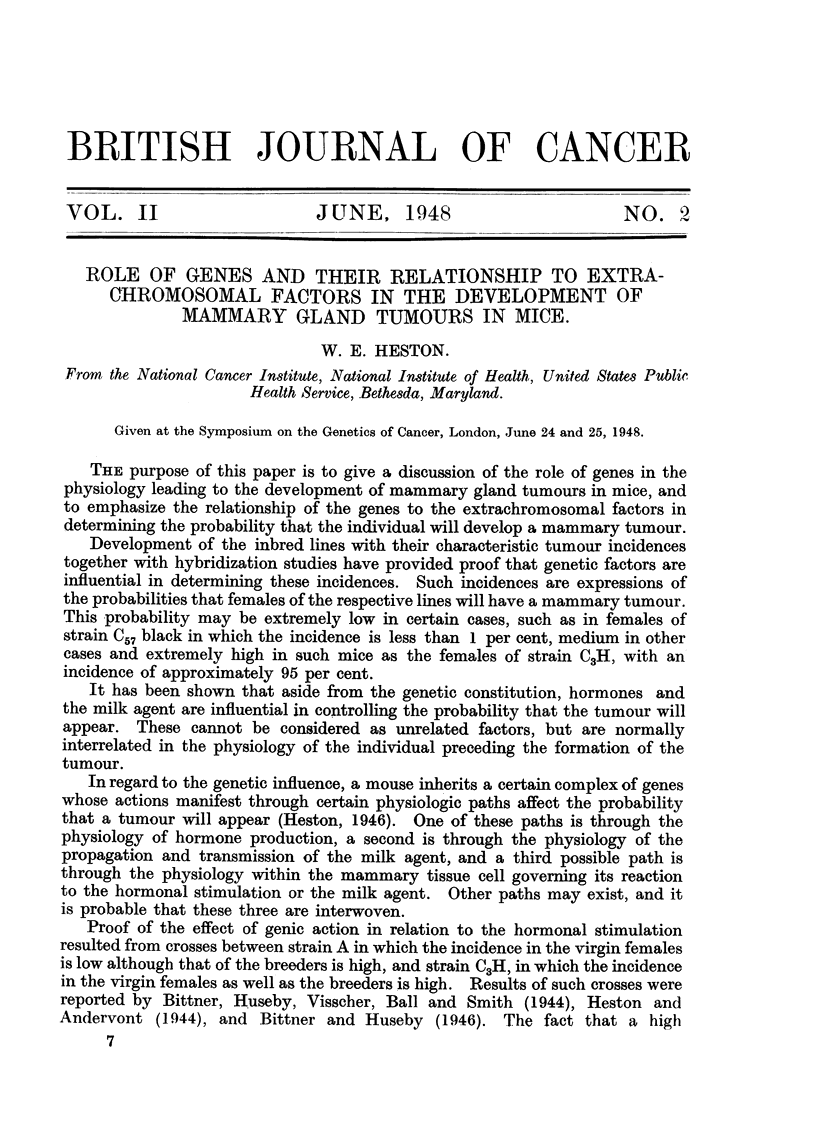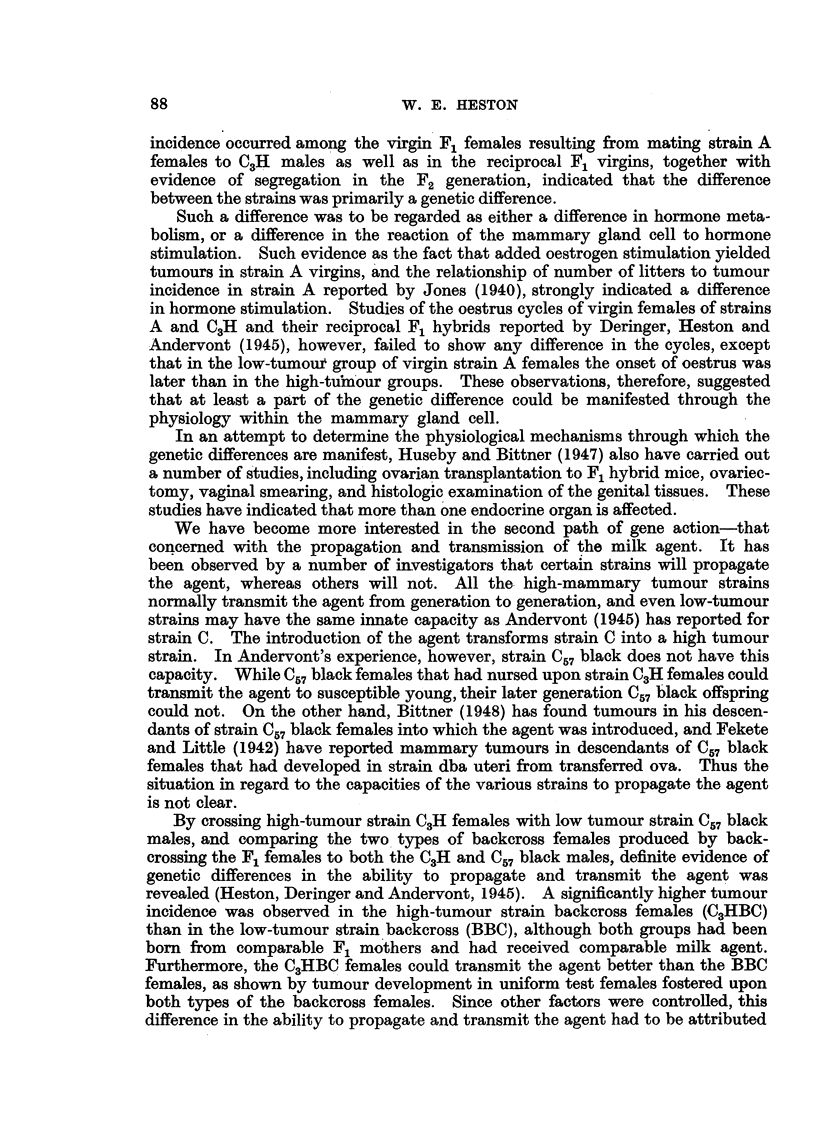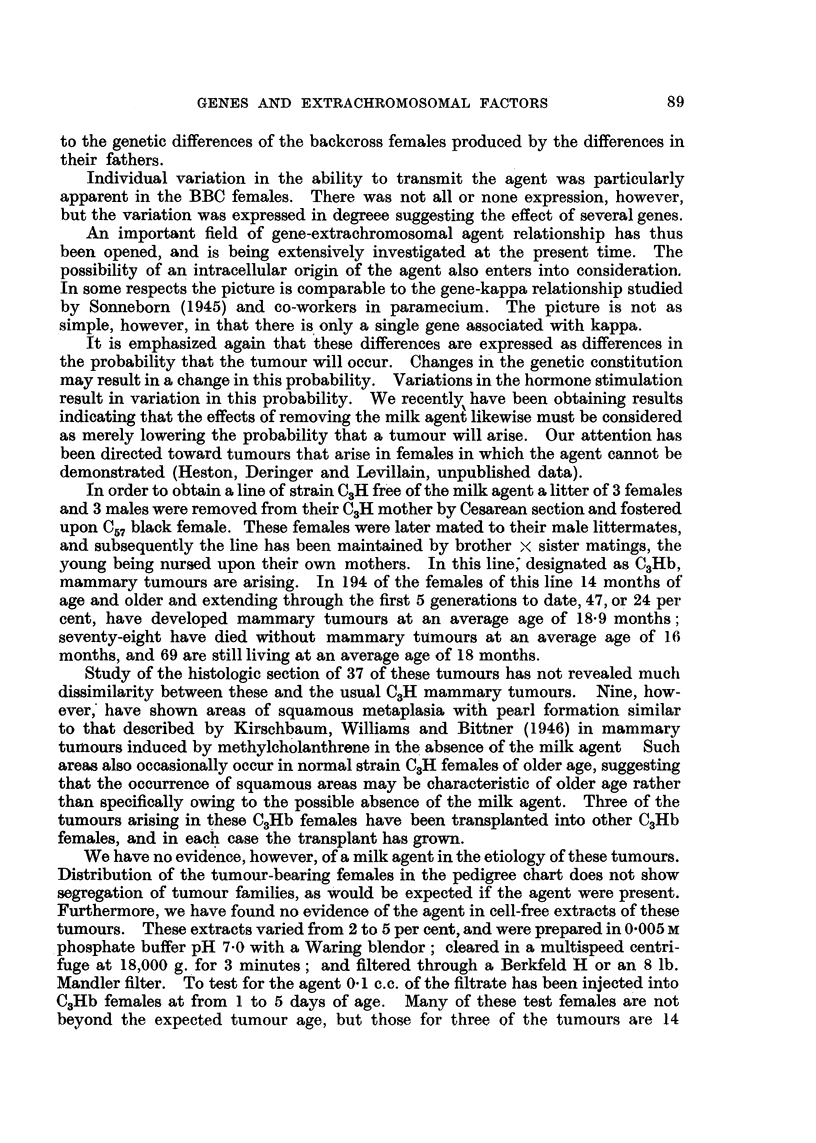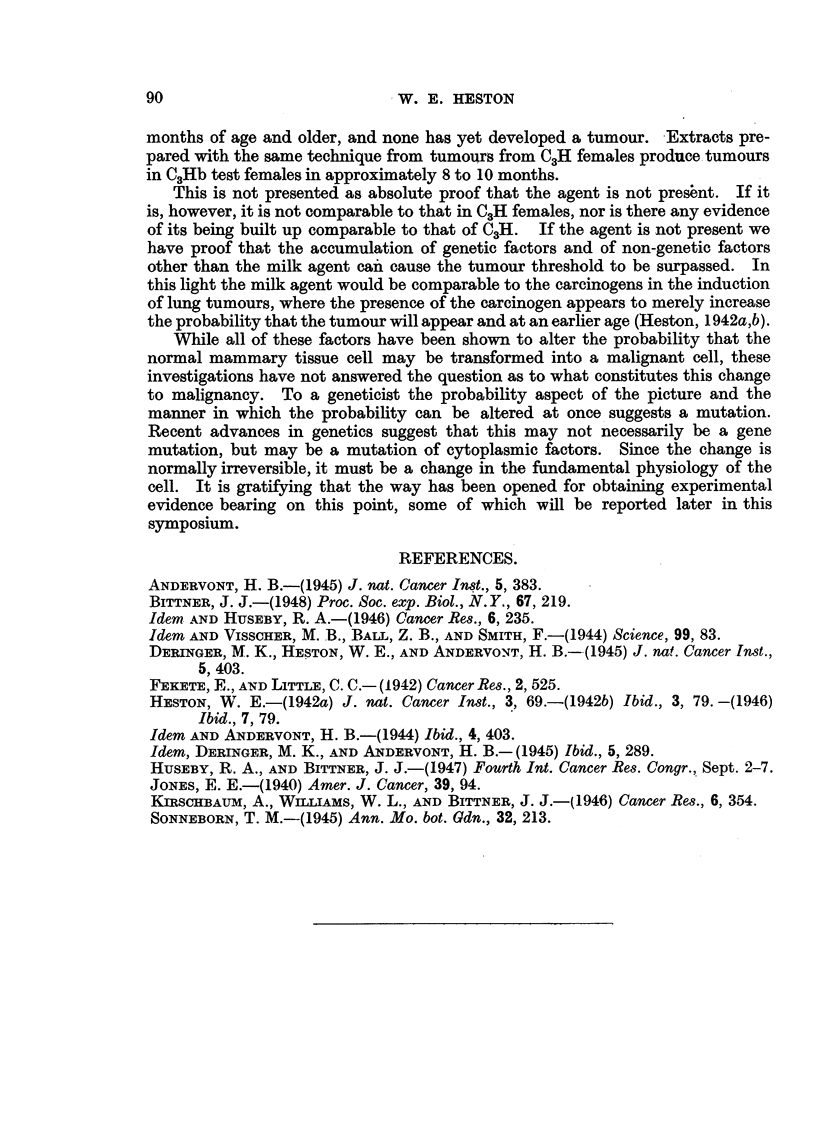# Role of Genes and their Relationship to Extrachromosomal Factors in the Development of Mammary Gland Tumours in Mice

**DOI:** 10.1038/bjc.1948.11

**Published:** 1948-06

**Authors:** W. E. Heston


VOL. II JUNE, 1948 NO. 2

### ROLE OF GENES AND THEIR RELATIONSHIP TO EXTRA-CHROMOSOMAL FACTORS IN THE DEVELOPMENT OF MAMMARY GLAND TUMOURS IN MICE.

W. E. HESTON.

*From the National Cancer Institute, National Institute of Health, United States Public Health Service, Bethesda, Maryland.*

Given at the Symposium on the Genetics of Cancer, London, June 24 and 25, 1948.

THE purpose of this paper is to give a discussion of the role of genes in the physiology leading to the development of mammary gland tumours in mice, and to emphasize the relationship of the genes to the extrachromosomal factors in determining the probability that the individual will develop a mammary tumour.

Development of the inbred lines with their characteristic tumour incidences together with hybridization studies have provided proof that genetic factors are influential in determining these incidences. Such incidences are expressions of the probabilities that females of the respective lines will have a mammary tumour. This probability may be extremely low in certain cases, such as in females of strain $C_{57}$ black in which the incidence is less than 1 per cent, medium in other cases and extremely high in such mice as the females of strain $C_3H$, with an incidence of approximately 95 per cent.

It has been shown that aside from the genetic constitution, hormones and the milk agent are influential in controlling the probability that the tumour will appear. These cannot be considered as unrelated factors, but are normally interrelated in the physiology of the individual preceding the formation of the tumour.

In regard to the genetic influence, a mouse inherits a certain complex of genes whose actions manifest through certain physiologic paths affect the probability that a tumour will appear (Heston, 1946). One of these paths is through the physiology of hormone production, a second is through the physiology of the propagation and transmission of the milk agent, and a third possible path is through the physiology within the mammary tissue cell governing its reaction to the hormonal stimulation or the milk agent. Other paths may exist, and it is probable that these three are interwoven.

Proof of the effect of genic action in relation to the hormonal stimulation resulted from crosses between strain A in which the incidence in the virgin females is low although that of the breeders is high, and strain $C_3H$, in which the incidence in the virgin females as well as the breeders is high. Results of such crosses were reported by Bittner, Huseby, Visscher, Ball and Smith (1944), Heston and Andervont (1944), and Bittner and Huseby (1946). The fact that a high incidence occurred among the virgin $F_1$ females resulting from mating strain A females to $C_3H$ males as well as in the reciprocal $F_1$ virgins, together with evidence of segregation in the $F_2$ generation, indicated that the difference between the strains was primarily a genetic difference.

Such a difference was to be regarded as either a difference in hormone metabolism, or a difference in the reaction of the mammary gland cell to hormone stimulation. Such evidence as the fact that added oestrogen stimulation yielded tumours in strain A virgins, and the relationship of number of litters to tumour incidence in strain A reported by Jones (1940), strongly indicated a difference in hormone stimulation. Studies of the oestrus cycles of virgin females of strains A and $C_3H$ and their reciprocal $F_1$ hybrids reported by Deringer, Heston and Andervont (1945), however, failed to show any difference in the cycles, except that in the low-tumour group of virgin strain A females the onset of oestrus was later than in the high-tumour groups. These observations, therefore, suggested that at least a part of the genetic difference could be manifested through the physiology within the mammary gland cell.

In an attempt to determine the physiological mechanisms through which the genetic differences are manifest, Huseby and Bittner (1947) also have carried out a number of studies, including ovarian transplantation to $F_1$ hybrid mice, ovariectomy, vaginal smearing, and histologic examination of the genital tissues. These studies have indicated that more than one endocrine organ is affected.

We have become more interested in the second path of gene action—that concerned with the propagation and transmission of the milk agent. It has been observed by a number of investigators that certain strains will propagate the agent, whereas others will not. All the high-mammary tumour strains normally transmit the agent from generation to generation, and even low-tumour strains may have the same innate capacity as Andervont (1945) has reported for strain C. The introduction of the agent transforms strain C into a high tumour strain. In Andervont's experience, however, strain $C_{57}$ black does not have this capacity. While $C_{57}$ black females that had nursed upon strain $C_3H$ females could transmit the agent to susceptible young, their later generation $C_{57}$ black offspring could not. On the other hand, Bittner (1948) has found tumours in his descendants of strain $C_{57}$ black females into which the agent was introduced, and Fekete and Little (1942) have reported mammary tumours in descendants of $C_{57}$ black females that had developed in strain dba uteri from transferred ova. Thus the situation in regard to the capacities of the various strains to propagate the agent is not clear.

By crossing high-tumour strain $C_3H$ females with low tumour strain $C_{57}$ black males, and comparing the two types of backcross females produced by backcrossing the $F_1$ females to both the $C_3H$ and $C_{57}$ black males, definite evidence of genetic differences in the ability to propagate and transmit the agent was revealed (Heston, Deringer and Andervont, 1945). A significantly higher tumour incidence was observed in the high-tumour strain backcross females ($C_3HBC$) than in the low-tumour strain backcross (BBC), although both groups had been born from comparable $F_1$ mothers and had received comparable milk agent. Furthermore, the $C_3HBC$ females could transmit the agent better than the BBC females, as shown by tumour development in uniform test females fostered upon both types of the backcross females. Since other factors were controlled, this difference in the ability to propagate and transmit the agent had to be attributed to the genetic differences of the backcross females produced by the differences in their fathers.

Individual variation in the ability to transmit the agent was particularly apparent in the BBC females. There was not all or none expression, however, but the variation was expressed in degreee suggesting the effect of several genes.

An important field of gene-extrachromosomal agent relationship has thus been opened, and is being extensively investigated at the present time. The possibility of an intracellular origin of the agent also enters into consideration. In some respects the picture is comparable to the gene-kappa relationship studied by Sonneborn (1945) and co-workers in paramecium. The picture is not as simple, however, in that there is only a single gene associated with kappa.

It is emphasized again that these differences are expressed as differences in the probability that the tumour will occur. Changes in the genetic constitution may result in a change in this probability. Variations in the hormone stimulation result in variation in this probability. We recently have been obtaining results indicating that the effects of removing the milk agent likewise must be considered as merely lowering the probability that a tumour will arise. Our attention has been directed toward tumours that arise in females in which the agent cannot be demonstrated (Heston, Deringer and Levillain, unpublished data).

In order to obtain a line of strain $C_3H$ free of the milk agent a litter of 3 females and 3 males were removed from their $C_3H$ mother by Cesarean section and fostered upon $C_{57}$ black female. These females were later mated to their male littermates, and subsequently the line has been maintained by brother $\times$ sister matings, the young being nursed upon their own mothers. In this line, designated as $C_3Hb$, mammary tumours are arising. In 194 of the females of this line 14 months of age and older and extending through the first 5 generations to date, 47, or 24 per cent, have developed mammary tumours at an average age of 18·9 months; seventy-eight have died without mammary tumours at an average age of 16 months, and 69 are still living at an average age of 18 months.

Study of the histologic section of 37 of these tumours has not revealed much dissimilarity between these and the usual $C_3H$ mammary tumours. Nine, however, have shown areas of squamous metaplasia with pearl formation similar to that described by Kirschbaum, Williams and Bittner (1946) in mammary tumours induced by methylcholanthrene in the absence of the milk agent Such areas also occasionally occur in normal strain $C_3H$ females of older age, suggesting that the occurrence of squamous areas may be characteristic of older age rather than specifically owing to the possible absence of the milk agent. Three of the tumours arising in these $C_3Hb$ females have been transplanted into other $C_3Hb$ females, and in each case the transplant has grown.

We have no evidence, however, of a milk agent in the etiology of these tumours. Distribution of the tumour-bearing females in the pedigree chart does not show segregation of tumour families, as would be expected if the agent were present. Furthermore, we have found no evidence of the agent in cell-free extracts of these tumours. These extracts varied from 2 to 5 per cent, and were prepared in 0·005 M phosphate buffer pH 7·0 with a Waring blendor; cleared in a multispeed centrifuge at 18,000 g. for 3 minutes; and filtered through a Berkfeld H or an 8 lb. Mandler filter. To test for the agent 0·1 c.c. of the filtrate has been injected into $C_3Hb$ females at from 1 to 5 days of age. Many of these test females are not beyond the expected tumour age, but those for three of the tumours are 14

months of age and older, and none has yet developed a tumour. Extracts prepared with the same technique from tumours from $C_3H$ females produce tumours in $C_3Hb$ test females in approximately 8 to 10 months.

This is not presented as absolute proof that the agent is not present. If it is, however, it is not comparable to that in $C_3H$ females, nor is there any evidence of its being built up comparable to that of $C_3H$. If the agent is not present we have proof that the accumulation of genetic factors and of non-genetic factors other than the milk agent can cause the tumour threshold to be surpassed. In this light the milk agent would be comparable to the carcinogens in the induction of lung tumours, where the presence of the carcinogen appears to merely increase the probability that the tumour will appear and at an earlier age (Heston, 1942*a*,*b*).

While all of these factors have been shown to alter the probability that the normal mammary tissue cell may be transformed into a malignant cell, these investigations have not answered the question as to what constitutes this change to malignancy. To a geneticist the probability aspect of the picture and the manner in which the probability can be altered at once suggests a mutation. Recent advances in genetics suggest that this may not necessarily be a gene mutation, but may be a mutation of cytoplasmic factors. Since the change is normally irreversible, it must be a change in the fundamental physiology of the cell. It is gratifying that the way has been opened for obtaining experimental evidence bearing on this point, some of which will be reported later in this symposium.

### REFERENCES.

Andervont, H. B.—(1945) *J. nat. Cancer Inst.*, **5**, 383.
Bittner, J. J.—(1948) *Proc. Soc. exp. Biol., N.Y.*, **67**, 219.
*Idem* and Huseby, R. A.—(1946) *Cancer Res.*, **6**, 235.
*Idem* and Visscher, M. B., Ball, Z. B., and Smith, F.—(1944) *Science*, **99**, 83.
Deringer, M. K., Heston, W. E., and Andervont, H. B.—(1945) *J. nat. Cancer Inst.*, **5**, 403.
Fekete, E., and Little, C. C.—(1942) *Cancer Res.*, **2**, 525.
Heston, W. E.—(1942*a*) *J. nat. Cancer Inst.*, **3**, 69.—(1942*b*) *Ibid.*, **3**, 79.—(1946) *Ibid.*, **7**, 79.
*Idem* and Andervont, H. B.—(1944) *Ibid.*, **4**, 403.
*Idem*, Deringer, M. K., and Andervont, H. B.—(1945) *Ibid.*, **5**, 289.
Huseby, R. A., and Bittner, J. J.—(1947) *Fourth Int. Cancer Res. Congr.*, Sept. 2–7.
Jones, E. E.—(1940) *Amer. J. Cancer*, **39**, 94.
Kirschbaum, A., Williams, W. L., and Bittner, J. J.—(1946) *Cancer Res.*, **6**, 354.
Sonneborn, T. M.—(1945) *Ann. Mo. bot. Gdn.*, **32**, 213.